# Genealogical data of Boer and Nubian goats in Mexico

**DOI:** 10.1016/j.dib.2020.105270

**Published:** 2020-02-08

**Authors:** N. Larios-Sarabia, J.A. Hidalgo-Moreno, R. Núñez-Domínguez, J.G. García-Muñiz, R. Ramírez-Valverde, H. Ben Zaabza

**Affiliations:** a University of Georgia, Department of Animal and Dairy Science, 30602, Athens, GA, USA; b Universidad Autónoma Chapingo, Departamento de Zootecnia, Posgrado en Producción Animal, km 38.5 carretera México-Texcoco, Chapingo, Estado de México, C.P. 56230, Mexico; c Natural Resources Institute Finland (Luke), FI-31600 Jokioinen, Finland

**Keywords:** Pedigree analysis, Inbreeding, Effective population size, Population structure

## Abstract

The pedigree file of the Boer and Nubian goat breeds in Mexico was constructed using the national database provided by the *Asociación Mexicana de Criadores de Ganado Caprino de Registro*. Field technicians routinely updated the goat national database by recording information from flocks participating in the performance-recording system. Information on animal identification number, parents, birth date, sex, breed, and farm of origin were used to undertake pedigree analyses using the ENDOG program (version 4.8). This paper presents a pedigree data file, tables and figures of characteristics of pedigree data, pedigree analyses, pedigree integrity, effective population size and genetic conservation index. The data can be used to estimate other population parameters, to monitor the genetic diversity of the Boer and Nubian goat breeds in Mexico, and also to design balanced breeding programs, maintaining genetic variation at reasonable levels and maximizing genetic progress in these populations.

**Table:** Specifications Table

Subject	Animal breeding and genetics
Specific subject area	Pedigree analyses to assess genetic diversity and population structure
Type of data	Tables and Figures
How data were acquired	During the 2018 genetic evaluation, genealogical information for the Boer and Nubian breeds was extracted from the performance-recording database provided by the *Asociación Mexicana de Criadores de Ganado Caprino de Registro*.
Data format	Raw, Analyzed, Filtered
Parameters for data collection	Field data was obtained from goat production units participating in the performance recording system of the *Asociación Mexicana de Criadores de Ganado Caprino de Registro*.
Description of data collection	Technical personnel from the *Asociación Mexicana de Criadores de Ganado Caprino de Registro* regularly visit purebred goat production units in Mexico. During such visits they physically evaluate the animals subjected to registration, they also collect and verify their pedigree and productive information. Animals that comply with the inspection parameters, genealogical and productive information, become part of the national breeding flock of their respective breed. Field data from all participating flocks is then concentrated and managed by the *Asociación Mexicana de Criadores de Ganado Caprino de Registro*, which then extends the animal registration certificates to individual goat breeders. The *Asociación Mexicana de Criadores de Ganado Caprino de Registro* provides the national database to *Universidad Autónoma Chapingo* for genetic evaluation purposes, and on a yearly basis, breeding values for growth traits are estimated and published.
Data source location	*Laboratorio de Evaluaciones Genéticas, Universidad Autónoma Chapingo, Departamento de Zootecnia, Posgrado en Producción Animal, km 38.5 carretera México-Texcoco, Chapingo, Estado de México*.
Data accessibility	Data is with this article.

**Table:** 

**Value of the Data** • Pedigree analysis of the Boer and Nubian goat breeds in Mexico assists stakeholders in monitoring the breeds' genetic diversity, and its outcome can be used to prevent significant loss of genetic diversity in breeding programs. • Monitoring the breeds' genetic diversity in a breeding program, through characterization of genetic structure and variability, may help maintain long-term genetic diversity and prevent genetic erosion and the presence of population bottlenecks. • Further, the data can be used to estimate other population parameters of genetic diversity for goats in Mexico, and to make other recommendations to maintain long-term genetic diversity and a sustainable genetic progress of these breeds.

## 1 Data

**Fig. 1:**
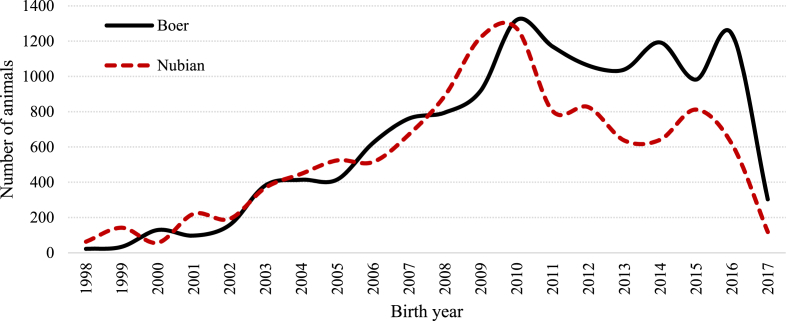
Number of animals registered by birth year for Boer and Nubian goats in Mexico.

**Fig. 2:**
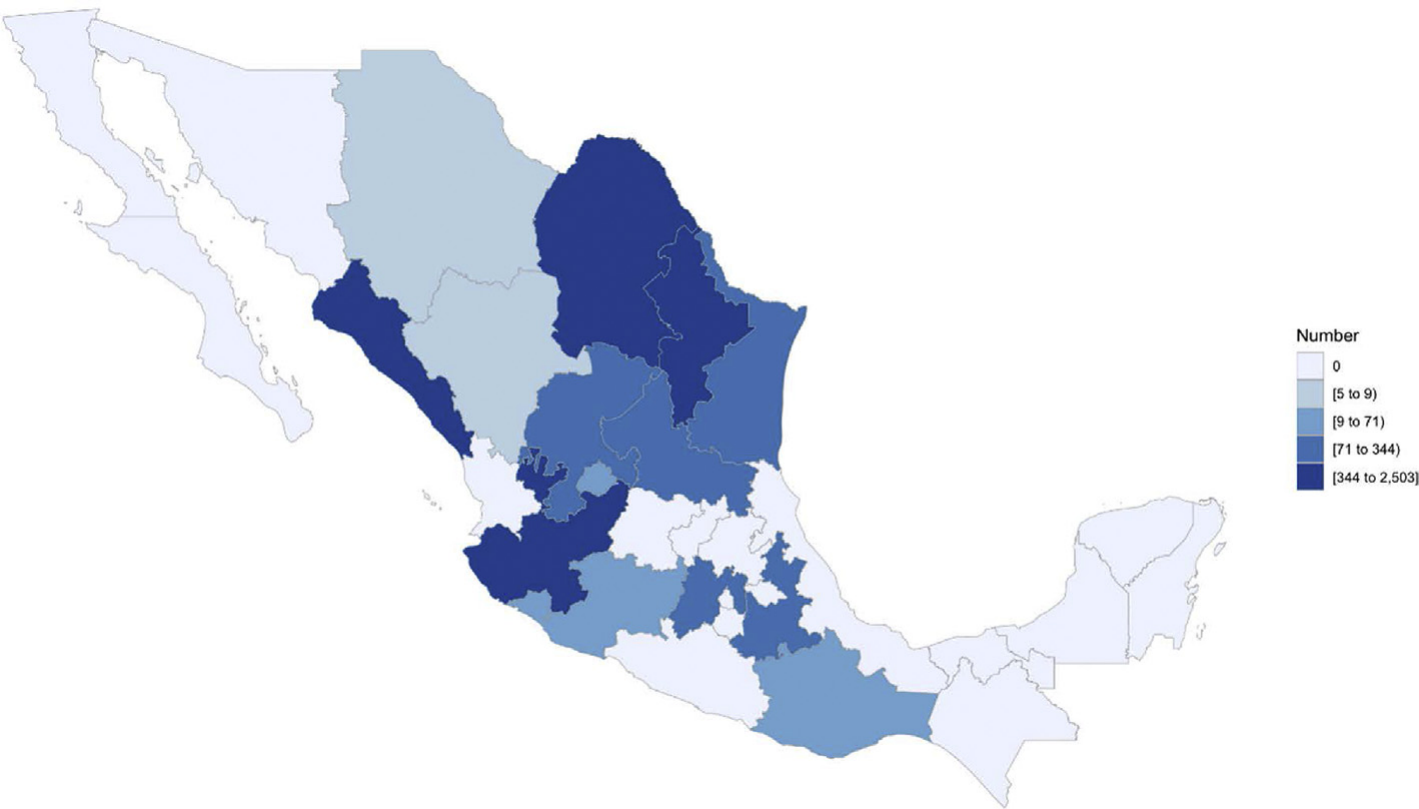
Population distribution of the reference population for Boer goats in Mexico.

**Fig. 3:**
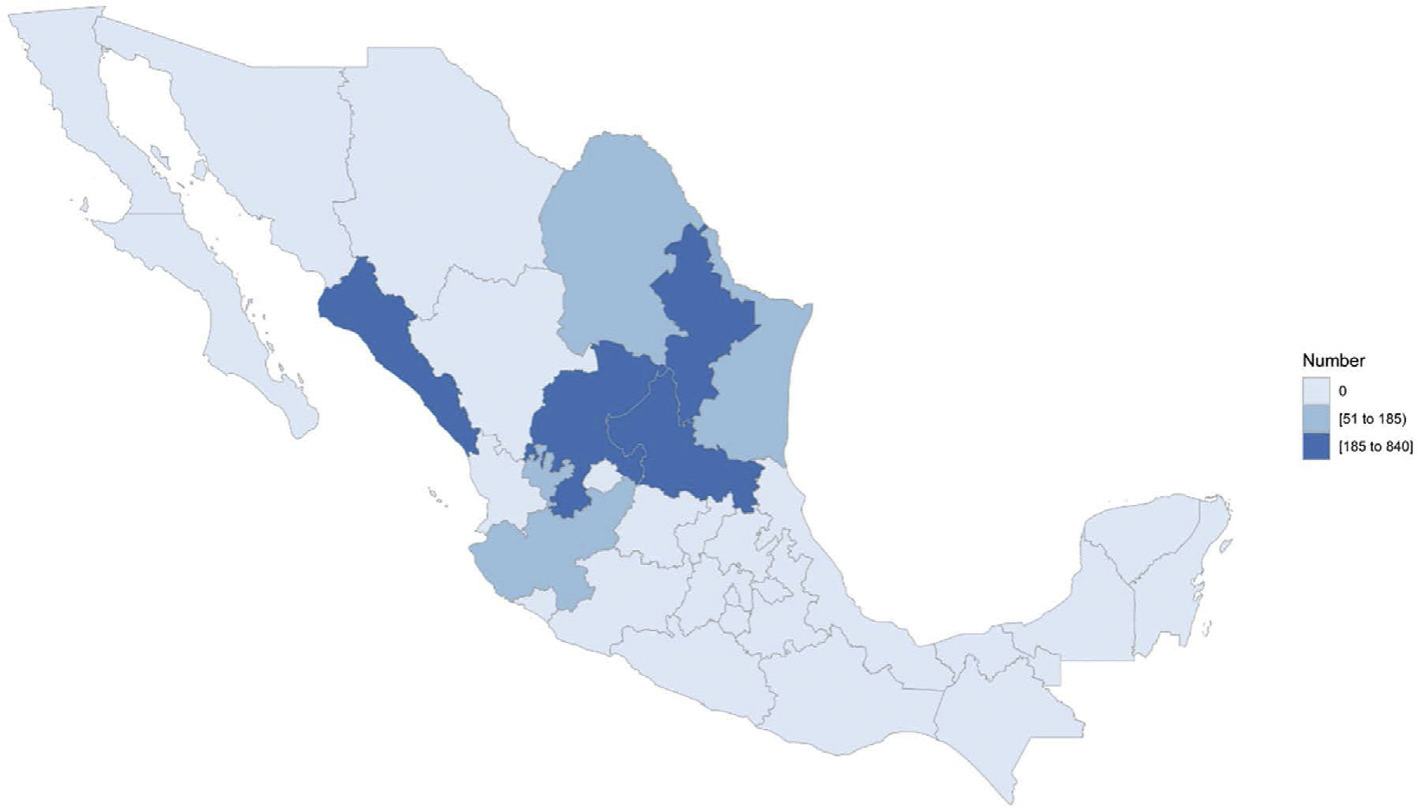
Population distribution of the reference population for Nubian goats in Mexico.

Individual records containing animal identification number, identification of parents, animal birth date, sex, breed, and farm of origin were obtained from the national database of the Boer and Nubian breeds in Mexico (Appendix A). Pedigree data for Boer and Nubian breeds comprised 141 and 99 flocks, respectively, that were distributed across 15 states in the country, but mainly distributed in the states of Nuevo León, San Luis Potosí, Sinaloa, Coahuila, Zacatecas and Jalisco (Fig. 2, Fig. 3). The majority of flocks were managed mostly on semi-intensive systems on native and cultivated pastures; supplementation with mineral salts and concentrate feed was provided in certain periods of the year depending on the availability of forage. Flocks’ reproduction took place mostly through controlled natural mating. Breeding was all-year-round, but the highest frequency of mating was from September to December; 90% of kidding were presented from January to April. Kids were naturally reared and suckling lasted three months, on average. The conformation of the pedigree file in both breeds predominantly represents the last ten years (Fig. 1). The characteristics of the pedigree files for the total and reference populations (kids born from 2013 to 2017 were considered as the reference population for both breeds) of Boer and Nubian goat breeds in Mexico are described in Table 1.

**Table 1:** Characteristics of pedigree data^a^ for the total and reference populations of Boer and Nubian goats in Mexico.

Parameter	Boer	Nubian
Total number of animals	18,947 (4758)	13,744 (2825)
Number of animals with progeny	7738 (566)	4837 (308)
Number of animals without progeny	11,209 (4192)	8907 (2517)
Number of animals with both parents known	12,290 (3824)	10,361 (2436)
Animals with both parents unknown	4004 (129)	1677 (27)
Number of animals with one parent known	2653 (805)	1706 (362)
Total number of bucks	2006 (77)	943 (22)
Total number of does	5732 (489)	3894 (286)
Number of animals with unknow buck	5061 (553)	2556 (278)
Number of animals with unknow doe	5600 (510)	2504 (138)
Average kids per buck	6.92 (17.27)	11.86 (14.82)
Maximum kids per buck	239 (94)	389 (64)
Average kids per doe	2.33 (2.07)	2.89 (2.06)

a Values without brackets correspond to the total population (in brackets are values for the reference population).

**Fig. 4:**
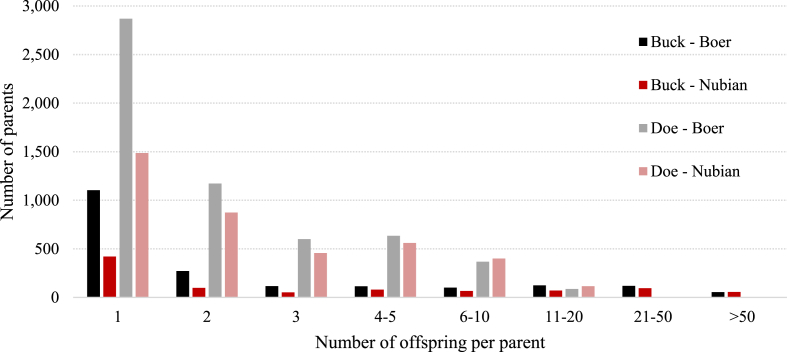
Frequency of parents by classes of number of offspring for Boer and Nubian goats in Mexico.

Breed differences in number of offspring per parent are illustrated in Fig. 4. Most of the bucks and does used for breeding purposes produce less than three kids.

**Fig. 5:**
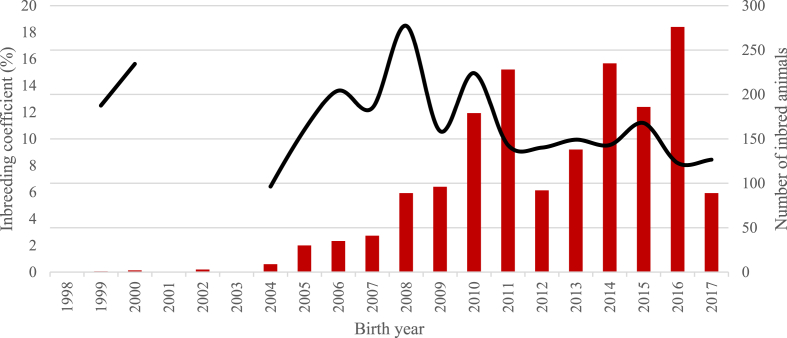
Number of inbred individuals (bars) and average inbreeding coefficient in inbred animals (line) for each year of birth for the Boer breed.

**Fig. 6:**
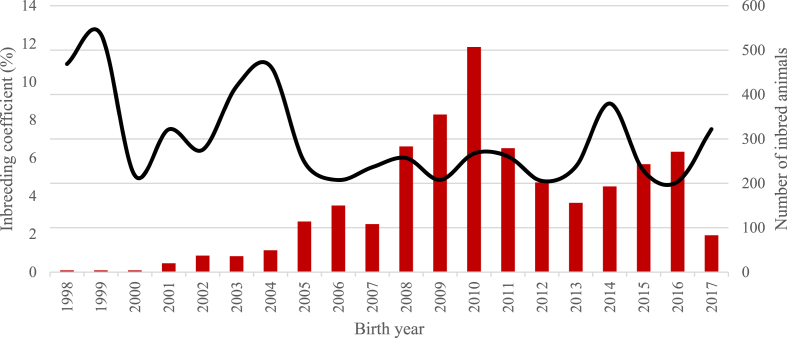
Number of inbred individuals (bars) and average inbreeding coefficient in inbred animals (line) for each year of birth for the Nubian breed.

The distribution of number of animals by inbreeding level and sex is presented in Table 2. The largest number of inbred animals were observed in the Nubian population, most of them were concentrated in the low levels of inbreeding (≤6.25%). On the other hand, for Boer, the largest number of inbred animals was concentrated in the class from 6.25 to 14.9%. In the last 8 (Boer) and 10 (Nubian) years there was an increase in the number of inbred animals (Fig. 5, Fig. 6). Nonetheless, the average inbreeding coefficient in inbred animals during this period was relatively stable.

**Table 2:** Number^a^ and sex of animals for different inbreeding levels for the total and reference populations in Boer and Nubian goats in Mexico.

Inbreeding level	Boer			Nubian		
	Males	Females	Total, per level	Males	Females	Total, per level
0.00%	6575 (1712)	10,590 (2122)	17,165 (3834)	4436 (900)	6154 (988)	10,590 (1888)
0.01–6.24%	250 (180)	314 (209)	564 (389)	863 (287)	1100 (328)	1963 (615)
6.25–14.99%	302 (164)	491 (189)	793 (353)	396 (92)	463 (101)	859 (193)
15.00–29.99%	107 (66)	301 (101)	408 (167)	104 (44)	218 (84)	322 (128)
30.00% or higher	4 (4)	13 (11)	17 (15)	4 (0)	6 (1)	10 (1)
Total, inbred animals	663 (414)	1119 (510)	1782 (924)	1367 (423)	1787 (514)	3154 (937)
Total	7238 (2126)	11,709 (2632)	18,947 (4758)	5803 (1323)	7941 (1502)	13,744 (2825)

a Values without brackets corresponding to the total population (in brackets corresponding to the reference population).

**Fig. 7:**
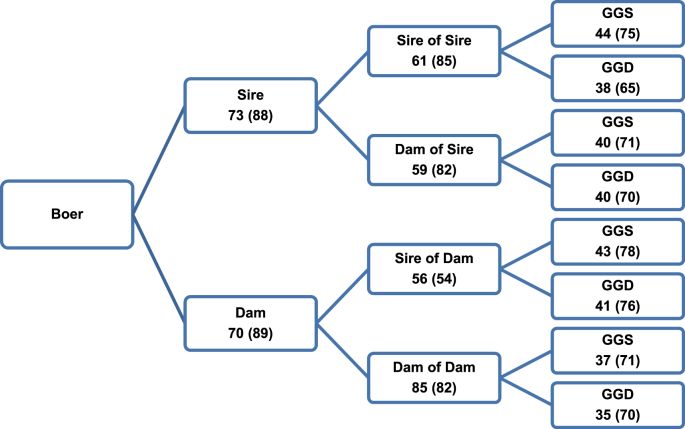
Percent of known ancestors for total and reference (in brackets) populations in the pedigree of the Boer breed. GGS = great-grand sire; GGD = great-grand dam.

**Fig. 8:**
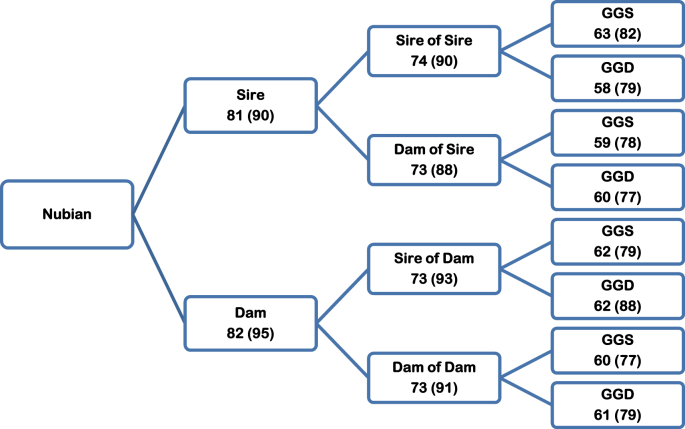
Percent of known ancestors for total and reference (in brackets) populations in the pedigree of the Nubian breed. GGS = great-grand sire; GGD = great-grand dam.

The pedigree structure showed that around 70% of the animals had identification of parents, almost 60% had identification of grandparents, and about 40% had identification of great-grandparents for the Boer breed (Fig. 7). For the Nubian breed, around 80% of animals had identification of parents, more than 70% had identification of grandparents and 60% had identification of great-grandparents (Fig. 8). In summary, the Pedigree Completeness Index in the first generation was 88.8 (Boer) and 92.6% (Nubian) for the reference population and 71.9 (Boer) and 81.6% (Nubian) for the total population. There were not big differences in the genealogy recording regarding mother or father side.

**Fig. 9:**
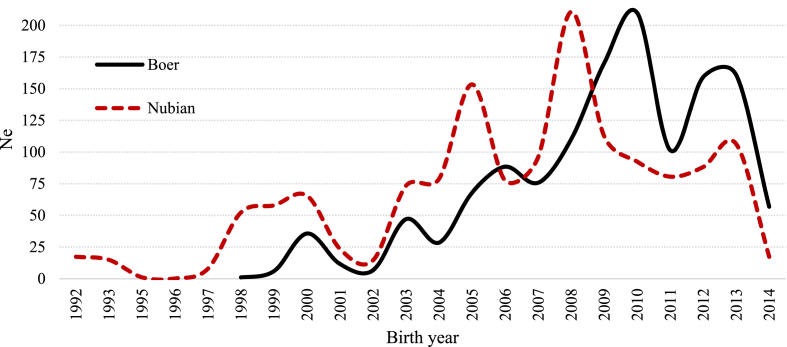
Effective population size (Ne) by birth year for Boer and Nubian goats in Mexico.

**Fig. 10:**
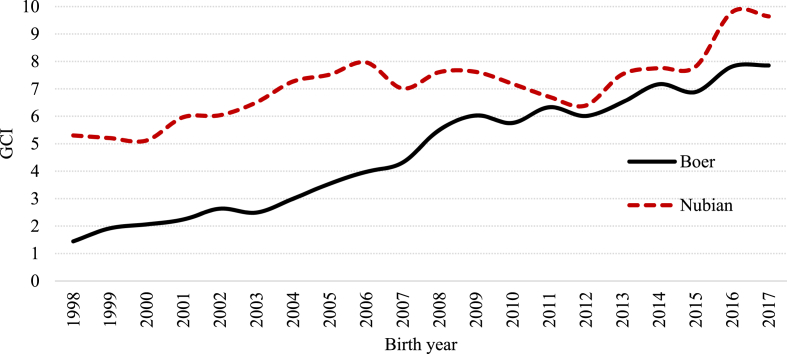
Evolution of average Genetic Conservation Index (GCI) through the studied period for the Boer and Nubian breeds in Mexico.

In general, since 2003 and 2005 (for Nubian and Boer, respectively) the effective population size showed an erratic pattern over the years. However, it remained over 50 (Fig. 9), indicating an acceptable level of genetic diversity for these two goat populations. On the other hand, for both breeds the average genetic conservation index (GCI) showed positive trends over the years (Fig. 10).

**Fig. 11:**
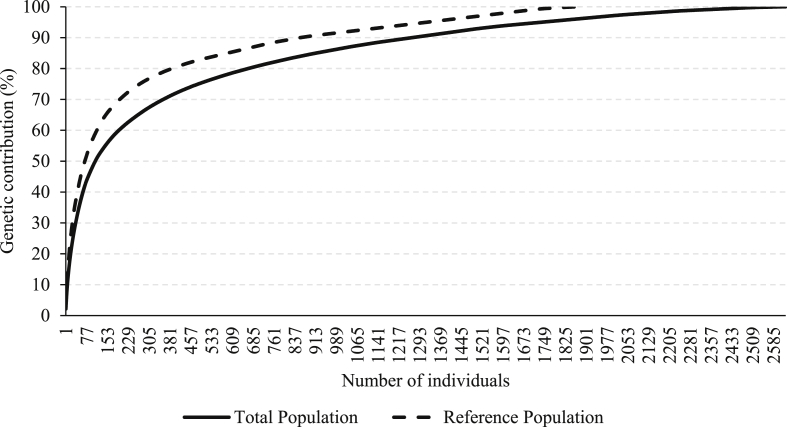
Cumulative genetic contribution of ancestors to the total and reference populations for the Boer breed.

**Fig. 12:**
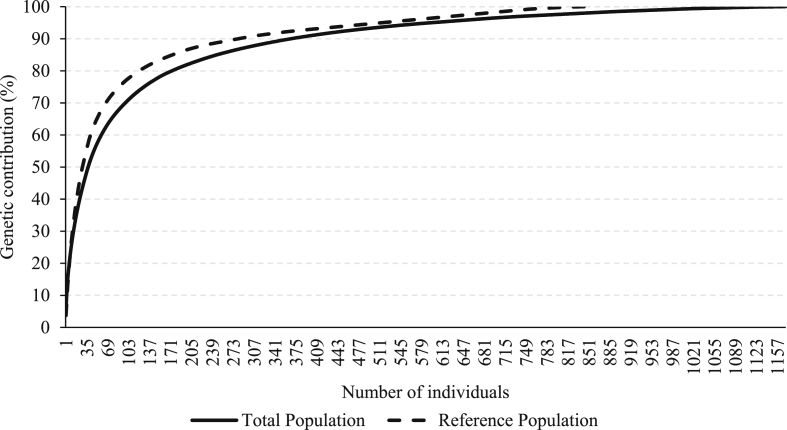
Cumulative genetic contribution of ancestors to the total and reference populations for the Nubian breed.

The following figures provide the number of most influential ancestors that explained the genetic diversity in the total and reference populations for the Boer (Fig. 11) and Nubian (Fig. 12) goat breeds. A less genetic contribution of ancestors was detected in the Nubian than in the Boer population.

## 2 Experimental design, materials, and methods

Pedigree analyses were performed using the ENDOG (version 4.8) program [1] with the procedures: pedigree content, founders, generations intervals, and offspring analysis. The results were obtained from the following output tables:

Midef: the inbreeding coefficient (F), relatedness coefficient (AR), and number of generations for each individual in the dataset.

GCI: the genetic conservation index computed for each animal as proposed by Alderson [2].

PediCont and PediContRef: the frequency of the contribution of given ancestor in the pedigree to the 5th parental generation for the whole pedigree or for a given reference population.

PedKnow and PedKnowRef: the completeness index for the whole pedigree or for a given reference population.

GenInterv and GeIntRef: average generation intervals and reproductive ages for each path parent–son, for the whole population or for a given reference population.

Ancestors and AncestorsRef: contribution to the population [3] for the whole population or for a predefined reference population.

NeOffs_Year: the estimates of effective population size by years.

## References

[1] Gutiérrez J.P., Goyache F. (2005). A note on ENDOG: a computer program for analysing pedigree information. J. Anim. Breed. Genet..

[2] Alderson G.L.H., Alderson L.J., Bodó I. (1992). A system to maximize the maintenance of genetic variability in small populations. Genetic Conservation of Domestic Livestock.

[3] Boichard D., Maignel L., Verrier É. (1997). The value of using probabilities of gene origin to measure genetic variability in a population. Genet. Sel. Evol..

